# Targeting epithelial-mesenchymal transition and cancer stem cells for chemoresistant ovarian cancer

**DOI:** 10.18632/oncotarget.9908

**Published:** 2016-06-09

**Authors:** Junli Deng, Li Wang, Hongmin Chen, Jingli Hao, Jie Ni, Lei Chang, Wei Duan, Peter Graham, Yong Li

**Affiliations:** ^1^ Cancer Care Centre, St George Hospital, Kogarah, NSW, Australia; ^2^ St George and Sutherland Clinical School, University of New South Wales (UNSW), Kensington, NSW, Australia; ^3^ Department of Gynecological Oncology, Henan Cancer Hospital, Zhengzhou, Henan, China; ^4^ Zhengzhou University, Zhengzhou, Henan, China; ^5^ School of Medicine, Deakin University, Waurn Ponds, Victoria, Australia

**Keywords:** ovarian cancer, EMT, CSC, chemoresistance, therapy

## Abstract

Chemoresistance is the main challenge for the recurrent ovarian cancer therapy and responsible for treatment failure and unfavorable clinical outcome. Understanding mechanisms of chemoresistance in ovarian cancer would help to predict disease progression, develop new therapies and personalize systemic therapy. In the last decade, accumulating evidence demonstrates that epithelial-mesenchymal transition and cancer stem cells play important roles in ovarian cancer chemoresistance and metastasis. Treatment of epithelial-mesenchymal transition and cancer stem cells holds promise for improving current ovarian cancer therapies and prolonging the survival of recurrent ovarian cancer patients in the future. In this review, we focus on the role of epithelial-mesenchymal transition and cancer stem cells in ovarian cancer chemoresistance and explore the therapeutic implications for developing epithelial-mesenchymal transition and cancer stem cells associated therapies for future ovarian cancer treatment.

## INTRODUCTION

Ovarian cancer (OC) is the leading cause of mortality from cancers of female reproductive tract in the world. Epithelial ovarian cancer (EOC) accounts for nearly 90% of all ovarian malignant diseases. EOCs fall into four main subtypes: serous, mucinous, endometrioid, and clear cell. In 2012, OC accounted for 151,900 deaths worldwide and there were 238,700 patients diagnosed with OC, according to the latest GLOBOCAN estimates [1]. OC is highly curable at stage I when it is confined to the ovaries, with an expected 5-year survival rate of 89% [2]. However, due to the absence of specific symptoms and the lack of an effective screening strategy, approximately 75% of women present at an advanced stage disease, where the cancer has spread within peritoneal cavity and the overall survival (OS) rates are only 17-36% [2].

For the early stage OC patients, surgery can completely remove tumor and then give patients a full recovery. For the advanced stage OC patients, cytoreductive surgery followed by platinum/taxane acts as a standard therapy, resulting in a 75% high initial response rate [3]. Although some patients may represent repeatedly sensitive to platinum, most patients will ultimately develop tumor recurrence and succumb to chemoresistant disease. Thus, improved targeting therapies and chemosensitization strategies are essential for reducing the mortality of this devastating malignancy.

Chemoresistance in cancer chemotherapy is a complicated process and affected by many factors. Although the exact mechanisms of OC chemoresistance and metastasis are still unclear, accumulating evidence supports that epithelial-mesenchymal transition (EMT) and cancer stem cells (CSCs) play critical roles in the development of resistance to chemotherapy, tumor relapse and metastasis of OC patients [4–7]. Understanding the mechanisms of chemoresistance will help develop novel therapies to improve OC patients' survival. In this review, we focus on the roles of EMT and CSCs in OC chemoresistance, and explore the EMT/CSC-based therapies for future OC treatment.

## EMT IN OVARIAN CANCER CHEMORESISTANCE

### EMT in OC metastasis

EMT is a process by which epithelial cells assume mesenchymal characteristics, facilitating migration through the extracellular matrix and settlement in areas of new organ formation during embryogenesis. This cell switch is known to be integral in development, wound healing and stem cell behavior, and contributes pathologically to fibrosis and cancer progression [8]. EMT occurs through down-regulation of E-cadherin, Cytokeratins, ZO-1, Claudins, Occludin, Laminin-1, Entactin, MUC-1, and the microRNA (miR-200 family), and up-regulation of the transcription factors Snail1, Snail2, Twist, Zeb1 and Zeb2/SIP1, E47, KLF8, E2.2, Goosecoid, LEF-1, and FoxC2, as well as N-cadherin, Vimentin, Fibronectin, miR-10b, and miR-21 [9–11]. In addition, EMT was reported to be induced by multiple signals, including growth factors, the Wnt/β-catenin signaling pathway, integrins, Notch transcription factors, prostaglandin E2, Cyclooxygenase-2, and hormones [9, 12].

During cancer progression, EMT appears to promote dissemination of cells from the tumor mass [13] and facilitate tissue invasion by regulating the production of matrix metalloproteases and altering cytoskeletal organization [14]. EMT plays an important role in OC metastasis. It was reported that EMT was induced by transforming growth factor-β (TGF-β), epidermal growth factor (EGF), hepatocyte growth factor (HGF) and endothelin-1 (ET-1) in OC cell lines [15]. Kajiyama and colleagues found that high level expression of Twist1 is associated with the FIGO stage as well as positive peritoneal cytology and predicts poor clinical outcomes in patients with clear cell carcinoma (CCC) of the ovary, suggesting that Twist1 may play a critical role in the metastatic process of CCC of the ovary [16]. Similarly, by comparing 54 cases of OC and paracancerous tissues, it was found that up-regulation of Twist is correlated with OC metastasis [17]. In another study, analysis of 174 primary tumors and 34 metastases of OC by immunohistochemistry (IHC) demonstrated a reduced E-cadherin expression and an increased expression of Snail are significantly associated with peritoneal metastasis and both the progression-free survival (PFS) and OS in patients with OC [18]. Zeb1, an important transcriptional suppressor of EMT, was found to be positively correlated with migration ability in SKOV-3 and HO8910 EOC cell lines, and the down-regulation of Zeb1 with shRNA) in SKOV-3 cells could significantly decrease tumor growth in mice xenograft [19]. These data support the significant role of EMT in OC progression, indicating targeting EMT holds promise to prevent OC progression.

### EMT in ovarian cancer chemoresistance

Increasing evidence demonstrates that there is a close relationship between EMT and chemoresistance in OC. Treatment with chemotherapeutics *in vitro* can induce chemoresistance and EMT. It was reported that continuous exposure to increasing doses of paclitaxel induced chemoresistance as well as EMT, and enhanced metastasis potential in EOC cells (NOS-2, TAOV and SKOV-3) [20]. Similarly, Latifi et al observed that cisplatin induced Twist1 expression in OVCA443 EOC cell line, with increased cell migration [21]. After treatment by carboplatin, the SKOV-3 EOC cells were demonstrated triggering both EMT and chemoresistance [22].

EMT markers and transcription factors are in correlation with chemoresistance in OC. In one study, up-regulation of EMT-related transcription factors Snail, Slug, Twist2 and Zeb2 in gene level and Snail, Slug, Vimentin in protein level was found in cisplatin resistant EOC cell line A2780-cis compared with cisplatin sensitive EOC cell line A2780 using gene expression and proteomic analysis, respectively [23]. Using 100 fresh advanced-stage ovarian serous carcinoma effusions, Davidson et al analyzed 10 EMT and CSC protein markers including E-cadherin, N-cadherin, P-cadherin, Zeb1, HMGA2, Rab25, CD24, NCAM (CD56), Sox11 as well as Vimentin, and identified Vimentin and Zeb1 as markers of poor chemoresponse in metastatic serous ovarian carcinoma effusions [24]. It was also found that reversal of EMT by down-regulating EMT makers can restore the chemosensitivity in OC. For example, Haslehurst et al found that, by reducing expression of Snail and Slug, the mesenchymal phenotype was largely reduced and cells were re-sensitized to cisplatin [23]. These findings demonstrate EMT has a critical role in OC chemoresistance, and inhibiting or reversing EMT could be a good choice in the treatment of OC.

The potential mechanisms of EMT in OC chemoresistance are still not fully uncovered. Accumulating evidence from preclinical and human tissue studies indicates that several important signaling pathways may contribute to OC chemoresistance via EMT, resulting in tumor metastasis and recurrence after chemotherapy. Different EMT-related signaling pathways associated with OC chemoresistance are summarized in Table 1. In Kurrey's study, both Snail and Slug were shown to impose acquisition of the CSC-like phenotype and chemoresistance in OC cells by overcoming p-53 mediated apoptosis [25]. Yue et al reported that hyperactive EGFR/STAT3 signaling promoted EMT during OC cisplatin resistance development [26]. By studying the molecular profiles from 23 stage III-IV OC biopsies at primary surgery, it was found that the activation of EMT by the TGF-β pathway is a signature indicative of resistance to platinum-based chemotherapy [5]. In another study, it was found that Notch3 activation induces EMT and attenuates carboplatin-induced apoptosis which is associated with inhibition of carboplatin-induced ERK phosphorylation in OVCA429 cells [27], indicating that Notch3 is associated with OC carboplatin resistance.

**Table 1:** The EMT-related signaling pathways in OC chemoresistance

Pathway	Experiment approach	EMT markers	Chemodrugs used in OC chemoresistance	Reference
p53 mediated apoptosis pathway	*in vitro* cell lines	Snail, Slug	paclitaxel	[25]
EGFR/Stat3 pathway	*in vitro* cell lines*; in vivo* animal models; and human tissues	Vimentin	cisplatin	[26]
TGF- β pathway	Human tissue	Zeb1	carboplatin and taxol	[5]
Notch3/ERK pathway	*in vitro* cell line	E-cadherin, Snail, Slug, SMA	carboplatin	[27]

**Figure 1:**
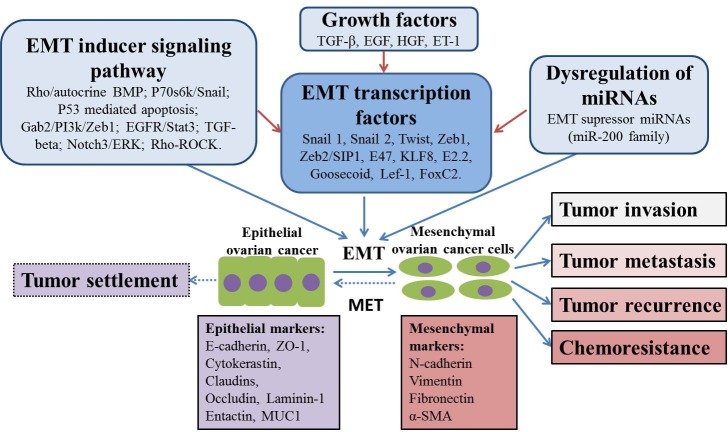
Putative mechanisms of EMT in ovarian cancer chemoresistance and progression EMT process plays an important role in tumor invasion, metastasis, recurrence and chemoresistance. This process is potentially regulated by different mechanisms, including EMT inducer signaling pathways, EMT transcription factors, dyregulation of miRNAs as well as several kinds of growth factors. In addition, MET may also happen during tumor progression in order to allow tumor growth and colonization, resulting in tumor settlement.

In addition to signaling pathways, microRNAs (miRNAs) also play a significant role in EMT in OC chemoresistance, among which miR-200 family is the most important one. The aberrant expression of miR-200 family (miR-200a, miR-200b, miR-200c, miR-141 and miR-429) in OC and its involvement in EMT were well-demonstrated [28], illustrating the importance of miR-200 family in OC chemoresistance through promoting EMT process. Using a well-characterized OC tissue achieve (*n* = 72), it was demonstrated that patients without complete response (CR) to paclitaxel-based chemotherapy had lower miR-200c levels than patients with CR, additionally, low miR-200 family (miR-200c, miR-141, and miR-429) expression had a trend toward poor PFS [29]. In another study, by analyzing the differences between biopsies from primary surgery and second surgery for relapse after several lines of chemotherapy (SCR) of 23 stage III-IV OC patients, the median expression levels of miR-200 family was observed to be down-regulated nearly two-fold in SCR group compared with those in primary surgery group, and the up-regulation of Zeb1 parallels the turn-off of miR-200 [5]. In a recent study, it was shown that paclitaxel resistant OVCAR-3/CP and MES-OV/CP EOC cell lines displayed a strong EMT phenotype, with marked decreases in expression of miR-200c and miR-141 in OVCAR-3/CP, and down-regulation of all five members of miR-200 family in MES-OV/CP [30]. Following inhibition of miR-200c and miR-141 in parental OVCAR-3 cells, EMT was triggered and rendered the cells resistant to paclitaxel and carboplatin, while the infection of paclitaxel resistant variants, OVCAR-3/TP cells with retroviral particles carrying the miR-200ab429 and 200c141 clusters triggered a partial mesenchymal to epithelial transition, further indicating the important role of miR-200c and miR-141 in OC chemoresistance and EMT [30]. The putative mechanisms of EMT in OC chemoresistance are shown in Figure 1.

All findings suggest that EMT is closely linked with OC metastasis, chemoresistance and progression, which is regulated by different signaling pathways and microRNAs, and that identification of main signaling pathways and new miRNA associated with EMT is undoubtedly an important step to develop new modalities to prevent metastatic, recurrent OC and improve the clinical outcomes of OC patients.

### Targeting EMT in OC treatment

Due to serious side effects of chemotherapy, targeted therapy plays a very important role in the current OC treatment. Accumulating evidence indicates that targeting EMT is a new developing research area and holds promise for chemoresistant OC patients in the future.

Developing cancer cell signaling pathway treatment is an important area for EMT-targeting therapy. In OVCA433 EOC cell line and tumors collected from advanced-stage EOC patients, ERK2 signaling pathway was found to be critical to cisplatin-induced EMT and CSC phenotypes. It was reported that the ERK inhibitor U0126 could block cisplatin-induced ERK2 activation, and sensitize the residual cells to increase cell death in response to cisplatin *in vitro* and inhibit cisplatin-induced migration in zebrafish embryos *in vivo* [21]. Hyperactive EGFR and the Jak-Stat3 pathways were found to be the key mediators of the EMT process associated with cisplatin resistance in cisplatin resistant EOC cell lines, and the combination of ZD1839 (EGFR inhibitor) or S3I-201 (Stat3 inhibitor) with cisplatin could sensitize cisplatin-resistant EOC cells *in vitro* and intra-peritoneal mouse xenografts *in vivo* [26]. Du et al found that EMT was related to the paclitaxel resistant A2780/PTX EOC cells, and the application of the PI3K inhibitor LY294002 could reverse the EMT phenotype and restore sensitivity to paclitaxel in A2780/PTX, indicating the therapeutic target of PI3K signaling pathway which is associated with EMT [31].

Epigenetic aberrations, including DNA methylation, histone modifications, and miRNA dysregulation also contribute to the EMT in OC. Therefore, reversing these changes has significant benefits as alternatives to traditional cytotoxic therapy. For example, epigenetic silencing of SFRP5 was observed to lead to oncogenic activation of the Wnt pathway and contribute to OC chemoresistance through the Twist-mediated EMT and AKT2 signaling, and the overexpression of SFRP5 inhibited EMT, down-regulated AKT2 and sensitized OC cells to chemotherapy, indicating the treatment promise of target reactivation of epigenetically silenced gene [32]. Trichostatin A, a histone deacetylase inhibitor or 5-aza-CdR, a DNA methyltransferase inhibitor, in combination with low-dose cisplatin significantly inhibited spheroid formation and tumorigenicity via the suppression of EMT and pluripotency of OC cells, both *in vitro* and *in vivo*. Most importantly, both epigenetic modifiers increased the sensitivity of the highly chemoresistant EOC cell line HEY to cisplatin, suggesting their promising application in OC chemoresistance treatment [33].

miRNAs have also shown their treatment promise in OC. Zhu et al demonstrated that miR-186 was detected to be associated with EMT and cisplatin-resistance in EOC cell lines (ACRP, C13* and OVCAR3/DDP) and human EOC tissues (cisplatin-sensitive tumors vs cisplatin-resistant tumors), and the introduction of miR-186 to EOC cells led to reduction of Twist1 as well as EMT, and rendered the cells more sensitive to cisplatin *in vitro* and *in vivo* [34]. Similarly, it was reported that miR-506 is associated with better therapy response and PFS and OS in EOC by analyzing The Cancer Genome Atlas (ACGA) (*n* = 486) and Bagnoli (*n* = 130) datasets [35]. As a result, miR-506 sensitizes EOC cells to chemotherapy and inhibits EMT-mediated metastasis.

In terms of the application of EMT marker targeting, Chiu et al illustrated that FOXM1 was a critical regulator of the EMT, stemness, and chemoresistance in EOC cells, and the combination of FOXM1 inhibitor thiostrepton and cisplatin inhibited the expression of EMT related markers (OCT4, Nanog, Notch-1) in chemoresistant A2780CP70 cells and growth of ovarian xenograft tumors in a subcutaneous (s.c) mouse model, indicating the promising application of EMT targeted therapy for future EOC treatment [36].

Some other small molecular inhibitors have also demonstrated the ability to reverse EMT and potentially increase chemosensitivity in OC. Using a gold nanoparticles (AuNPs), Xiong et al reversed cisplatin-induced chemoresistance by inhibiting EMT *in vitro* using EOC cell lines (A2780, OVCAR5, SKOV3-ip), and sensitized orthotopically implanted ovarian tumors to a low dose of cisplatin *in vivo* on athymic nude female mice [37]. Targeting EMT with different approaches in OC is shown in Table 2.

**Table 2:** Targeted therapy for reversing EMT in the treatment ovarian cancer chemoresistance

Approach for EMT targeting	Chemodrug used in combination	Target	Effect	Targeting site mechanism	Experiment type	Reference
Thiostrepton	cisplatin	FOXM1	Chemosensitivity↑, stemness↓	EMT-related marker (FOXM1)	*in vitro, in vivo*	[36]
UO126	cisplatin	ERK	Chemosensitivity↑, migration↓	EMT-related ERK pathway	*in vitro, in vivo*	[21]
S31-201	cisplatin	Stat3	Chemosensitivity↑	EMT-related Jak-Stat3 pathway	*in vitro, in vivo*	[26]
ZD1839	cisplatin	EGFR	Chemosensitivity↑	EMT-related EGFR pathway	*in vitro, in vivo*	[26]
LY294002	paclitaxel	PI3K	Chemosensitivity↑	EMT-related PI3K pathway	*in vitro, in vivo*	[31]
Zibotentan	cisplatin or taxol or paclitaxel	ET_A_R	Chemosensitivity↑, invasiveness↓	EMT-related ET_A_R/b/are-stin-1 pathway	*in vitro, in vivo*	[38]
SFRP5 expression vector	cisplatin or taxol or etoposide	SFRP5	Chemosensitivity↑	Epigenetic therapy	*in vitro, in vivo*	[32]
Trichostatin A	cisplatin	histone deacety-lase	Chemosensitivity↑	Epigenetic therapy	*in vitro, in vivo*	[33]
5-aza-CdR	cisplatin	DNA methylt-ransferase	Chemosensitivity↑	Epigenetic therapy	*in vitro, in vivo*	[33]
miRNA-186	cisplatin	Twist 1	Chemosensitivity↑	Epigenetic therapy	*in vitro, in vivo*	[34]
miRNA-506	cisplatin and olaparib	RAD51 and Snail1	Chemosensitivity↑, metastasis↓	Epigenetic therapy	*in vitro, in vivo*	[35]
AuNPAs (gold nanoparticle)	cisplatin	N/A	Chemosensiti-vity↑, metastasis↓	Targeted therapy	*in vitro, in vivo*	[37]

**Notes:** N/A: not available; ↑: increase; ↓: reduce

All findings support that targeting EMT is a bright research area to prevent OC chemoresistance and it is worthwhile developing combination approaches in the future to improve current modalities. However, we need to realize that although these agents can reverse OC chemoresistance for combination therapy, they have very broad activities or targeting signaling pathways that are not limited to the EMT targeting. Therefore, when choosing these agents for combination therapy, we should maximise their anti-EMT effects and avoid their side effects.

## CSCS IN OVARIAN CANCER CHEMORESISTANCE

### CSC models

Although there exists a long-lasting debate regarding the origin of CSCs, it is widely accepted that tumors are composed of phenotypically and functionally heterogeneous cells and CSCs are only a small subset of tumors cells. The stochastic model, hierarchy model and the dedifferentiation model are the three major theories as to how CSCs arises.

The earlier CSC model is a static one. According to stochastic model, tumor cells are biologically equivalent but behave variably due to stochastic influences (intrinsic and extrinsic factors). The core of this theory is that behaviors of tumor cells cannot be predicted and every tumor cell is thought to have the potential to behave the activity of CSCs [39]. The hierarchy model, which is the most universal accepted hypothesis, supports that tumors consist of distinct cell classes with differing functional abilities and behaviors on the basis of different intrinsic characteristics. Based on this model, CSCs are the only subpopulation possessing self-renewal and giving rise to non-tumorigenic progenies that make up the bulk of tumor [39]. However, data emerging in the last couple of years has revised the model to a dynamic one, where the hierarchical feature of the CSCs turns out to be more transient than once thought. That is, new progenies acquire the ability of self-renewal through de-differentiation of progenitor cells, as well as reversal of terminally differentiated cells [40]. The implications of CSCs and their offspring gaining self-renewal suggest the necessity to evolve current cancer treatments to target both bulk terminal differentiated cells and those with self-renewal potential [41].

### The putative CSC markers in ovarian cancer

Accumulating evidence indicates that CSCs have close relationship with OC progression, metastasis, therapeutic resistance and tumor recurrence. The concept of CSCs has opened new areas of research in carcinogenesis, but has more immediate translational potential of uncovering new treatment targets.

Ovarian CSCs (OCSCs) have been isolated from established OC cell lines, ascites, and primary and metastatic tumors [42–45]. They share several characteristics with normal stem cells, including the ability to form anchorage-independent spherical aggregates, express stem cell markers, undergo membrane efflux, form clones in culture and in addition, exhibit enhanced tumor-forming ability [46]. A number of cell surface markers have proved useful for the isolation of subsets enriched for OCSCs including CD44, CD133, CD117, CD24, ALDH1A1 and EpCAM.

CD44 is a cell-surface glycoprotein involved in cell-cell interactions, cell adhesion and migration. The multiple protein isoforms are encoded via a single gene by alternative splicing and are further modified by a range of post-translational modifications (PTMs) [47]. CD44 was well documented to be a common CSC marker in many cancers such as breast cancer, head and neck squamous cell carcinoma, pancreatic cancer, colon cancer, as well as OC, and proved to be correlated with therapeutic resistance. Zhang et al isolated and characterized ovarian cancer-initiating cells (OCICs) from primary ovarian tumors using CD44 and CD117 antibodies, which were fully capable of re-establishing their original tumor hierarchy *in vivo*, indicating that both CD44 and CD117 positive cells are OCSCs (Zhang, Balch et al. 2008). Besides, they also found that CD44^+^/CD117^+^ cells had increased chemoresistance to taxane and platinum-based chemotherapy as well as the ability to self-propagate [42]. Similarly, Alvero and colleagues showed that CD44^+^ cells were enriched in the ascites of OC patients, and the isolated CD44^+^ cells in mouse xenograftes gave rise to tumors with both CD44^+^ and CD44^−^ cells, suggesting that those CD44^+^ cells can differentiate and self-renew with the feature of cancer-initiating cells, in addition, CD44^+^ EOC cells were demonstrated to be more resistant to paclitaxel and carboplatin compared with CD44^−^ cells.[48].

CD117, also known as c-Kit or stem cell growth factor receptor, is a proto-oncogene encoded by the KIT gene. It is a type of tyrosine kinase receptor involved in cell signal transduction and has been shown to be involved in apoptosis, cell differentiation, proliferation, and cell adhesion [49]. CD117 was found to have high expression in OC cells [45]. Interestingly, using cancer cells isolated from human primary OC tissues and ascites, Luo and his colleagues demonstrated that CD117^+^ OC cells appeared to be highly tumorigenic than CD117^−^ OC cells as it only took approximately 10^3^ cells to be able to self-renew, differentiate, and regenerate tumor in mouse models, and CD117 expression was statistically correlated with resistance to conventional chemotherapy [50]. Another study also reported that the Wnt/β-catenin pathway which plays an important role in the development of chemoresistance is activated by CD117 in SKOV-3 and HEYA8 EOC cell lines [51], further confirming the importance of this marker in OC chemoresistance.

CD133, a pentaspan membrane glycoprotein, has been identified as a CSC marker for various cancers [52]. In EOC, CD133 has emerged as one of the most promising CSC markers based on *in vitro* cell lines, *in vivo* animal xeongrafts and human primary tumor experiments. It was reported that CD133^+^ A2780, PEO1 EOC cells generate both CD133^+^ and CD133^−^ daughter cells, exhibiting enhanced resistance to platinum-based therapy *in vitro* and sorted CD133^+^ OC cells from A2780 line formed more aggressive tumor xenografts at a lower inoculum than their CD133^−^ progenies on BALB/cAnNCr-nu/nu mice [43]. Curley et also found that tumor-derived CD133^+^ cells from human primary OC tissues have an increased tumorigenic capacity and are capable of recapitulating the original heterogeneous tumor in NOD/SCID mice compared with CD133^−^ cells [44].

CD24, the cell surface protein, is highly expressed in many human cancers [53]. It was reported that CD24 could be used as a CSC marker in nasopharyngeal carcinoma [53], pancreatic cancer [54] and OC [55]. Gao et al isolated cancer cells from human primary ovarian tumor specimens and identified a sub-population defined by CD24 phenotype. Their experiments demonstrated that, compared with CD24^−^ cells, CD24^+^ cells possesses stem-cell like characters with relative quiescence, self-renewal, differentiation, chemoresistance *in vitro*, and a xenograft injection of 5000 CD24^+^ cells produced tumors in nude mice, while injection of an equal number of CD24^−^ cells failed to do so [55]. Using a transgenic murine model of OC, Burgos-Ojeda et al recently found CD24^+^ OC cells were enriched for cancer-initiating cells, and CD24^+^ cells have increased tumor sphere-forming capacity and play a role in tumor migration and metastasis [56]. However, Ment et al reported that the CD44^+^/CD24^−^ phenotype in OC cells demonstrateCSC-like properties of enhanced differentiation, invasion, and resistance to chemotherapy and correlation to clinical endpoints with increased risk of recurrence and shorter PFS duration in patients with OC [57]. Gunjal et al also found that CD24^−^ cells, if expanded from a singly sorted cell, may give rise to cells containing all of the markers, including CD24 [58]. Based on these findings, CD24 cell surface marker in CSC phenotype fluctuates with tumor environment and cell expansion, suggesting that the role of CD24 in OCSC remains plastic and inconclusive, and further investigation is needed to elucidate its role.

ALDH enzymes belongs to a family of evolutionarily conserved enzymes comprised of 19 isoforms that are localized in the cytoplasm, mitochondria or nucleus. Generally, ALDHs are generated by a wide variety of metabolic processes and are responsible for oxidizing aldehydes to carboxylic acids [59]. ALDH was initially used to isolate CSCs in leukemia, based on the increased ALDH activity using the aldeflour assay [60]. Landen et al for the first time isolated putative CSCs in OC by high ALDH activity, and showed that high ALDH expression predicts poor outcome in OC patients [61]. Later on, Silva et al demonstrated that as few as 11 enriched ALDH^+^CD133^+^ CSCs isolated from human ovarian tumors were sufficient to initiate tumors in mice and that the presence of ALDH^+^CD133^+^ cells in debulked primary tumor specimens correlated with reduced disease-free survival (DFS) and OS in OC patients, indicating that both ALDH and CD133 can be used as a functionally significant set of markers to identify OCSCs [62]. Liu et al. recently demonstrated that elevated ALDH expression was associated with poor prognosis in OC patients using meta-analysis [63]. Similarly, Mizuno et al also found high ALDH1 expression levels in human OC tissues are related to advanced stage in clear cell carcinoma cases and ALDH1 expression significantly reduced PFS. They also found that ALDH^+^ CSCs might have increased Nrf2-induced antioxidant scavengers, which lower ROS level relevant to chemoresistance in ovarian clear cell carcinoma [64]. In another study, high level expression of Lgr5 and ALDH1 in primary EOC was found to correlate with advanced tumor stage and grade as well as poor prognosis of the patients [65]. These data suggest ALDH is an OCSC marker associated with EOC progression and chemoresistance.

EpCAM (epithelial cell adhesion molecule), is a glycosylated, 30~40 kDa type I transmembrane protein, which was reported to be expressed on essentially all human adenocarcinoma, and is part of the signature of CICs in numerous solid tumors as well as normal stem cells [66]. EpCAM has been used as an important OCSC marker. Katia et al selected a CD44^+^/CD24^+^/EpCAM^+^/E-cadherin^−^ subpopulation of cells from EOC cells (SKOV-3 and OVCAR-5) using flow cytometry, and found that this small population of cells (less than 1%) has increased colony formation and shorter tumor-free intervals on a mouse s.c xenograft model *in vivo* after limited dilution, showing initiating tumor cell properties, and is resistant to both doxorubicin and cispation therapies [67]. By evaluating the expression of EpCAM at both RNA and protein levels in 4 normal fresh-frozen ovaries and 96 EOC biopsies (50 primary ovarian carcinomas, 34 metastatic, and 12 recurrent ovarian tumors, respectively), Bellone et al. found that EpCAM was significantly expressed in EOC tissues compared to the normal ovary tissues, and metastatic/recurrent tumours were found to express higher levels of EpCAM than primary ovarian carcinomas. More interestingly, a high surface expression was also found in 100% (5/5) of the chemoresistant ovarian carcinoma cell lines derived from OC patients studied by flow cytometry [68]. These results indicate that EpCAM has significantlly clinical significance for targeting chemoresistant recurrent OC.

Using existing CSC makers to define OCSC is an important step to uncover OC chemoresistant mechanisms, find useful therapeutic targets and develop new treatment modalities to cure metastatic, recurrent OC. However, none of these current CSC markers are exclusively expressed by OC tissues, highlighting that it is imperative to use combinatorial makers or delineate more specific markers and techniques to detect OCSCs.

In addition to cell surface OCSC markers, side population (SP) cells also play an important role in identification of OCSCs. SP cells can be separated by using the fluorescent DNA-binding dye such as Vybrant^®^ DyeCycleTM Violet [69], and Hoechst 33342 [70], depending on the expression of ATP-binding cassette transporters (ABC transporters), or the unique pattern of cell growth in specific cell culture medium for sphere formation [71]. The SP cells were normally demonstrated to be highly enriched for CSC markers and endowed with regenerative capacities. For instance, after several passages, purified sphere-forming cells were isolated and found to express various stem cell markers (stem cell factor, Notch-1, Nanog, ABCG2, and Oct-4)[72].

The studies from different groups have identified and isolated SP of OC cells with typical “CSC phenotype” (ability to self-renew, developing tumors in very small numbers~100 cells, resistance to therapy) [42, 55, 73–74]. In a systematic screen of 6OCcell lines, Boesch et al demonstrated that the SP of EOC cells defines a heterogeneous compartment exhibiting CSC characteristics and the exact identity of the CSC is still disguised by the high complexity of the CSC-containing compartment, suggesting that further functional studies are needed to determine whether a single cellular subset can unambiguously be defined as CSC or whether multiple stem cell-like cells with different properties coexist [69]. Yasuda et al use of SP and aldehyde dehydrogenese bright (ALDH^Br^) overlapping population is a promising approach to isolate highly purified CSCs/CICs from OC cells and demonstrated this population exhibited higher tumor-initiating ability than that of SP cells or ALDH ^Br^ cells alone, enabling initiation of tumor with as few as 100 cells and showing higher sphere-forming ability, cisplatin resistance, adipocyte differentiation ability and expression of SOX2 than those of SP/ALDH^Low^, MP (main population)/ALDH^Br^ and MP/ALDH^Low^ cells [75]. SP population was reported to be increased in paclitaxel-resistant OC cell lines (2008/PX24, KF28TX, TU-OM-1 TX) but not increased in cisplatin-resistant cell lines (C13, KFr13, TU-OM-1 CDDP), suggesting SP cells are specially associated with paclitaxel resistance [76] Using Hoechst 33342 dye efflux, Szotek et al identified and characterized SP cells from two distinct genetically engineered mouse OC cell lines (MOVCAR7 and 4306) with the capacity for self-renewal and production of heterologous non-SP progeny, and found that SP cells showed a higher tumor forming ability and chemoresistance than non-SP cells [70]. These results indicate SP population has an important role in OC chemoresistance.

Due to the importance of CSCs and the limitation of the current techniques to effectively detect CSCs, some new approaches are required to separate SP cells possessing CSCs properties for an in-depth investigation. All putative OCSC markers, origin and tumorigenicity in mice are shown in Table 3.

**Table 3:** Putative OCSC markers and tumorigenicity

Marker	Cell source	Minimum cell number required for tumorigenicity in mice	Reference
CD44	Primary human ovarian tumors	10^2^ (CD44^+^/CD117^+^)	[42]
	Ascites from OC patients	N/A	[48]
CD117	Primary human ovarian tumors and ascites	10^3^ (CD117^+^)	[50]
CD133	A2780 cell line	10^3^ (CD133^+^)	[43]
	Primary human ovarian tumors	10^4^ (CD133^+^)	[44]
CD24	Primary human tumors	5×10^3^ (CD24^+^)	[55]
ALDH	A2780 cell line	10^3^ (ALDH^+^/CD133^−^), 30 (ALDH^+^/CD133^+^)	[62]
EpCAM	SKOV-3, OVCAR-5 cell lines	10^2^ (EpCAM^+^/CD24^+^/CD44^+^/E-cadherin^−^ from OVCAR-5)	[67]
SP (efflux Vybrant^®^ DyeCycleTM Violet)	A2780, A2780V, B2/92, B16/92, B17/92, IGROV1 cell lines	mice receiving 10^4^ SP cells succumbed to the tumor burden significantly earlier than did the NSP controls	[69]
SP (efflux Hoechst 33342)	MOVCAR7 and 4306 mouse OC cell lines	1.5×10^5^ SP at 10 weeks, 7.5 x10^5^SP at 7 weeks (MOVCAR 7 cell line)	[70]
SP (efflux Hoechst 33342)	PTX-resistant cell lines (2008/PX24, KF28TX, KFr13TX, and TU-OM-1 TX)	N/A	[76]
SP/ALDH^Br^	MCAS, HTBoA, OVCAR3, OVSAHO, HEC-1cell lines	10^2^	[75]

Notes: N/A: not available; NSP: non side population; SP: side population; OC: ovarian cancer; PTX: paclitaxel

### Significance of CSCs in ovarian cancer chemoresistance

The CSC theory has offered a potential explanation for the relapse and resistance that occur in many tumors including OC after therapy [77]. CSCs prossess the capability to recapitulate a heterogeneous tumor upon transplantation and may produce tumors through self-renewal and differentiation into multiple cell types. These CSCs may provide a reservoir of cells that cause tumor recurrence after therapy [78]. CSCs embody the refractory nature observed among many cancers: very competent initial tumor establishment, extremely aggressive metastatic nature, resistance to chemo- and radiotherapy, correlation with advanced disease and resistance to current therapies. Therefore, if CSCs survive after anti-cancer treatment, recurrence and metastasis are expected. Thus, investigation of CSCs has been a hot spot of basic cancer research and is rapidly expanding into many related aspects of cancer research, including chemo-sensitization.

Meng and colleagues demonstrated that the increased numbers of CD44^+^ cells from OC cell lines (SKOV-3, TOV112D and ES2) and patient ascites samples increased chemoresistance, and that patient ascites samples with > 25% CD44^+^ cells had significantly decreased median PFS (6 month vs 18 month, *P* = 0.01) as well as propensity to recur (83% vs 14%, *P* = 0.003) [57]. Through comparison of chemoresistant and chemosensitive primary OC tissue samples, Bonneau et al found that CD44 expression on tumor cells appeared to correlate with OS and could be used as a predictor of chemoresistance [79]. Similarly, by comparing the paired primary, metastatic, recurrent OC tissues from 26 individual patients, another study showed that both the metastatic and recurrent tissues expressed higher level of CD44 than the patient matched primary tumors, and a significant association was found between CD44 expression and both the DFS and OS. In the following *in vitro* and *in vivo* experiments, they also observed the overexpression of CD44 in chemoresistant cell lines (SKOV-3TR, OVCAR-8TR), and the knockdown of CD44 by shRNA could significantly increase the sensitivity of OC cells (OVCAR8) to paclitaxel [80].

Zhang et al found associations between CD133^+^ and higher grade ovarian tumors, advanced stage disease, and decreased response to chemotherapy in 400 OC primary tissue samples, demonstrating that CD133^+^ tumors are associated with decreased OS and shorter disease free interval [81]. In a meta-analysis, high CD133 expression level was also found to correlate with advanced tumor stages and a worse prognosis (reduced 2-year survival) in patients with OC [81]. Using a functional enrichment strategy, Chau et al separated a SP of cells from SKOV-3 and HEYA8 EOC cell lines and found that the SP cells showed high CD117 expression, and the inhibition of CD117 (c-Kit) with c-Kit knockdown or imatinib (a c-Kit kinase activity inhibitor) could significantly reduce resistance to chemotherapeutic drugs (cisplatin/paclitaxel). [51]. Based on the analysis of 64 primary high grade ovarian serous carcinoma and peritoneal metastasis, it was demonstrated that patients with high CD117 expression in tumor cells had significantly shorter DFS, high CD133 expression was correlated with shorter DFS and OS, and CD133 was proved to be an independent prognostic factor in the high grade serous carcinomas [82]. It was reported that in 65 advanced stage OC patients, more than 20% of ALDH1A1^+^ cells were found to be correlated with decreased PFS [61]. Zhu et al reported that overexpression of CD24 in EOC was an independent indicator associated with a low survival rate, increased metastasis, and decreased survival time [83]. These data support OCSCs have clinical significance for patients' survival and disease course.

**Figure 2:**
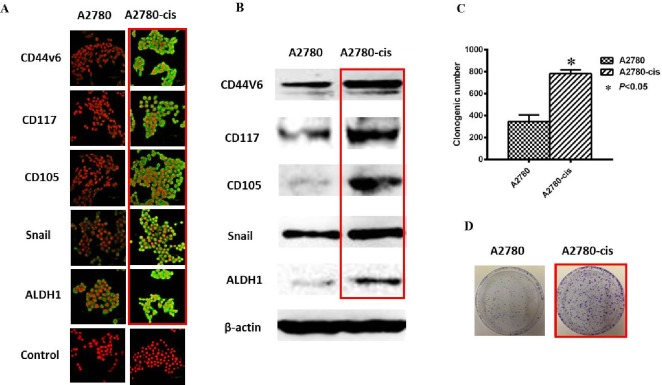
Increased CSC phenotype in EOC-cis resistant cells compared with EOC-control cells **A.** Representative immunofluorescence images are shown for increased expression of CSC related markers (CD44v6, CD117, CD105, Snail and ALDH1) in A2780-cis resistant cells compared with A2780-control cells. Nuclei were stained with PI. Magnification: all images x 600. **B.** Western blotting results were consistent with immunofluorescence staining results. β-actin was used as a loading control. **C.** Increased clone forming ability was found in A2780-cis resistant cells compared with A2780 cells (*P* < 0.05). **D.** Typical images for colony growth in A2780-cis and A2780 cells. The results were from 3 independent experiments (*n* = 3).

Increasing number of studies have demonstrated that CSCs may confer growth advantage and metastasis in chemoresistant ovarian tumors [43, 45]. Silva et al reported that isolated ALDH^+^CD133^+^ OCSC-like cells from human primary ovarian tumors showed increased chemoresistance compared to their parental cells and the presence of ALDH^+^CD133^+^ cells in debulked primary tumors correlated with reduced disease-free and OS in OC patients [62]. Cole et al observed that short-term chemotherapy to EOC cell lines (SKOV-3 and OVCA429) resulted in the purification of a subpopulation of cells overexpressing CSC-related markers (CD117, CD133, OCT4 and Nanog), and those CSC-like cells exhibited increased chemoresistance [84]. Abubaker et al also demonstrated that the chemotherapy-treated residual ovarian tumors were enriched in cells with CSC phenotypes on mice xenograft with HEY cells injection while the cells lacking these characteristics were eliminated both *in vitro* and *in vivo* [85]. We have recently found increased CSC phenotypes (CD44v6, CD117, CD105, Snail and ALDH1) and colony formation ability in a chemoresistant EOC cell line A2780-cis compared to their parental cell line A2780 (Figure 2). All these results suggest that chemoresistance has a close relationship with CSCs in OC, and the existence of CSCs may be the source of chemoresistance and recurrence.

### The potential mechanisms of CSCs in ovarian cancer chemoresistance

**Figure 3:**
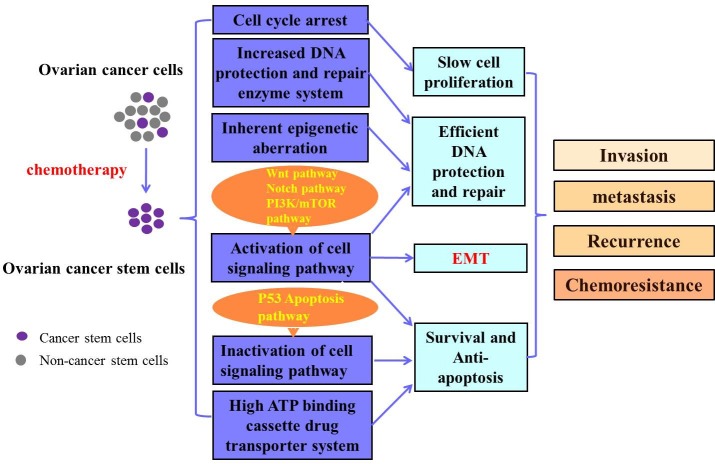
Putative mechanisms of CSCs in OC chemoresistance Ovarian cancer stem cells (OCSC) can be enriched after chemotherapy. The OCSCs may be related with several mechanisms including cell cycle arrest, increased DNA protection and repair enzyme system, inherent epigenetic aberration, the activation of some cell signaling pathways related to cell survival, inactivation of some cell signaling pathway correlated with cell death, as well as the high ATP binding cassette drug transporter system. These mechanisms contribute to ovarian cancer invasion, metastasis, recurrence and chemoresistance. EMT: epithelial mesenchymal transition.

The mechanism of CSCs in OC chemoresistance and recurrence is complex and not fully understood. It is possible that decreased chemotherapy responsiveness of CSCs may be partly due to the slow proliferation rate, cell cycle arrest, the high expression of ATP transporters, efficient DNA protection and repair mechanisms, the activation of some CSC-related signaling pathways, inactivation of cell death pathways, and the inherent epigenetic aberrations. The putative mechanisms of CSC in OC chemoresistance are shown in Figure 3.

It was reported that conventional chemotherapeutic treatment, which usually targets fast-dividing cells and acts in a cell-cycle specific manner, entitles CSCs the survival advantage because of their slow proliferation rate [86]. Gao et al found that CD24^+^ CSC cells were relatively quiescent and enriched at the S phase, leading to the chemotherapeutic resistance to cisplatin treatment compared with CD24^−^ OC cells from human ovarian tumor specimens [55]. Abubaker et al demonstrated that a short-term single exposure of chemotherapy induced surviving OC cells with a CSC-like profile which was independent of the type of chemotherapy and the associated cytotoxicity [87].

CSCs are known to possess highly elaborated efflux systems for cytotoxic agents, of which ABC family of membrane transporters are the most important ones. In return, there is strong collective evidence that increased expression and the activity of ABC family of membrane transporters, especially ABCG2, also correlates with cancer stem-like phenotype [88]. Ricci et al demonstrated that higher levels of ABCG2 efflux pump in OCSC-like cells were linked with increased resistance to taxol and VP16 therapy in OC cells which were obtained from primary ovarian carcinoma samples [89]. It was reported that Wnt/β-catenin-ABCG2 signaling pathway was activated and enhanced chemoresistance was observed in OCSCs, and β-catenin small interfering RNA (siRNA) reversed the drug sensitivity of OCSCs significantly [51]. Notch-1 and Nanog were also found to be co-upregulated with ABCG2 when ovarian tumour initiating cells were cultured under stem cell-selective conditions, accompanied by enhanced chemoresistance to the OC chemotherapeutics [42].

SP is also found to be related to OC chemoresistance via ABCG2. Kruger et al. demonstrated that SP cells from two murine carcinoma cell lines exhibited up-regulation of ABCG2 and stem cell markers Wnt-1 and Sca-1, increased resistance to chemotherapy, increased efflux of chemotherapeutic agents and increased ability to generate tumors *in vivo* [90].

Moreover, up-regulated DNA protection and repair and inactivation of apoptosis may also be responsible for chemoresistance in OCSCs. Srivastava et al reported that an elevated expression of DNA polymerase η (Pol η) was observed in OCSCs isolated from both OC cell lines and primary tumors, and down-regulation of Pol η enhanced the cisplatin-induced apoptosis in CSCs both *in vivo* and *in vitro* [91], indicating that CSCs may have intrinsically enhanced translation DNA synthesis. On the other hand, a study demonstrated that p53 protein aggregation was associated with the inactivation of the p53-mediated apoptosis and platinum resistance in OC cells with CSC properties [92].

In summary, there are many mechanisms involved in the OCSC-associated chemoresistance. Research efforts should be put in uncovering these mechanisms in the future in order to pave the way for OCSCs targeting therapy.

### Targeting CSCs for OC treatment

CSCs are implicated in cancer metastasis, recurrence, and therapeutic resistance. Targeting CSCs may possess many advantages by eradicating the root of tumor and managing their malignant behaviors. However, the data related with effective targeting strategies for CSCs are still limited until now. The current CSC targeting therapy in OC are mainly focused on the employment of OCSCs markers and the signaling pathways related to CSCs.

Imatinib (Gleevec), a clinical drug that blocks Abelson cytoplasmic tyrosine kinase (ABL), CD177, and PDGFR, demonstrated its inhibition potency on OCSCs. In this study, the combination of imatinib with cisplatin/paclitaxel showed higher efficiency against the regeneration of drug-resistant OCSCs compared to imatinib or cisplatin/paclitaxel alone [51], indicating the potential use of imatinib in the OCSCs target therapy for OC. However, in a phase II clinical trial, using a 400 mg dose of imatinib had no significant effect on PFS in recurrent EOC [93, 94]. In another phase II clinical trial, at a higher dose of 600 mg imatinib, no clinical response was seen [95]. In platinum-resistant, recurrent EOC and with tumors that were positive for CD177 and PDGFR, no response rate was found with a 400 mg dose of imatinib in a phase II clinical trial [96]. Overall, the response rate of Imatinib has been very modest in clinical studies. Skubitz et al reported that targeting CD133 directly with a dCD133KDELa monoclonal antibody (MAb) that recognizes a non-glycosylated region of CD133, could successfully inhibit tumor growth and progression in OC mouse model [97], further providing the possibility of applying OCSC markers for future treatment.

In addition, some signaling pathway inhibitors preferentially targeting CSCs are also useful in reversing chemoresistance. VS-5584, a dual inhibitor of mTORC1/2 and class I PI3-Kinase, was reported to be up to 30-fold more potent in inhibiting the proliferation and survival of CSCs compared with non-CSC in OC and other solid tumor cells, and most importantly, VS-5584 was also able to significantly repress tumor regrowth after chemotherapy in an immunodeficient OC mouse model [98]. In another study, using OC cell line HEY, Abubaker et al demonstrated that inhibition of the JAK2/STAT3 pathway by CYT387 suppressed the ‘stemness’ profile in chemotherapy-treated residual OC cells *in vitro* and animal xenograft models *in vivo*, leading to a reduced tumor burden [85]. Ibrutinib, a novel Bruton's tyrosine kinase (Btk) inhibitor, was found to be able to significantly reduce the expression of JAK2/STAT3 pathway which in turn suppressed the survival of cancer cells through Sox-2 and BCL-XL genes and restored chemosensitivity in cisplatin-resistant OC spheroids [99]. Furthermore, it was also reported that γ-secretase inhibitor GSI a Notch pathway inhibitor, was able to deplete CSCs and increase ovarian tumor sensitivity to platinum, and the combination of cisplatin and GSI was shown to effectively eliminate both CSCs and the bulk of tumor cells by enhancing the DNA-damage response, G2/M cell-cycle arrest, and apoptosis [100]. We have recently demonstrated the activation of the PI3K/Akt/mTOR signaling pathway is associated with EMT and CSCs in chemoresistant EOC cell lines and combination of cisplatin and a dual PI3K/mTOR inhibitor (BEZ235) could increase chemosensitivity (unpublished observation), suggesting the PI3K/Akt/mTOR pathway plays an important role in EOC chemoresistance.

Some emerging novel strategies have also demonstrated anti-CSC effects for improving OC chemoresistance. Donahoe et al demonstrated that mullerian inhibiting substance (MIS mimetic SP600125) preferentially inhibited chemotherapy-enriched CSC-like cells in human EOC cell lines including OVCAR-5, IGROV-1 and SKOV-3 and provided a new treatment paradigm [73]. SGI-110, a new DNA methytransferace inhibitor, was found to reduce stem cell property of OC in a low dose through inducing re-expression of differentiation-associated genes *HOXA10*, therefore decreasing tumor initiating capacity and re-sensitizing CSCs to platinum. Moreover, the maintenance treatment with SGI-110 after carboplatin was also shown to inhibit CSC growth and decrease tumor progression in animal models *in vivo* [101]. Wang et al also demonstrated a low-dose SGI-110 reduced the stem-like properties of ALDH^+^ EOC cells, including their tumor-initiating capacity, resensitized these OCSCs to platinum, and induced re-expression of a differentiation-associated gene in mRNA level (*HOXA10* mRNA) [101].

Wu et al recently reported that solanum incanum extract (SR-T100), containing the active ingredient solamargine, can down-regulate the expression of stem cell markers, including ALDH1, Notch1, and FoxM1, and reduce sphere formation in OC cells, increase the sensitivity of chemoresistant OC cells (A2780CP70) to cisplatin and paclitaxel *in vitro*. Furthermore, combination treatment using cisplatin and SR-T100 was more effective in inhibiting the tumor growth of A2780CP70 cells in mouse xenografts than either therapeutic alone, suggesting SR-T100 may have potential against chemoresistant OC cells [102]. Our group have developed aptamer-guided therapies such as aptamer-CD133 and aptamer-EpCAM conjugates for cancer treatments in preclinical studies and demonstrated low cytotoxicity, great binding affinity and penetration ability, and high efficacy for CSC targeting [103–104]. Aptamer-CD133 and aptamer-EpCAM hold promise for targeting OCSCs in the future research. Different inhibitors for OCSC-targeted therapy are shown in Table 4.

**Table 4:** Targeting CSC surface markers or CSC-associated signaling pathways in ovarian cancer treatment

Target	Inhibitor	Reference
CD117	Imatinib	[51]
CD133	dCD133KDEL	[97]
PI3K/mTOR pathway	VS-5584	[98]
JAK2/STAT3 pathway	Ibrutinib	[99]
JAK2/STAT3 pathway	CYT387	[85]
Notch pathway	GSI	[100]
DNA methytransferace	SGI-110	[101]

CSC markers are very dynamic and plastic during cancer progression. CSC phenotypes are regulated by tumor microenvironment. When designing a CSC targeting therapy, the CSC plasticity should be taken into consideration. It is important to determine the CSC expression at different stages of cancer. In addition, targeting several CSC markers may achieve a better clinical result than only targeting one CSC marker.

## CONCLUSIONS AND FUTURE DIRECTIONS

Chemotherapy remains an important modality for advanced OC treatment with ongoing efforts towards designing new treatment modalities and techniques, which continue to improve the survival and quality of life in OC patients. However, chemoresistance and tumor recurrence are the major challenges for the current treatment failure. It is increasingly clear that EMT and CSCs play important roles in OC metastasis and progression and are related with chemoresistance, and targeting EMT and CSCs or their corresponding pathways by gene therapy, antisense therapy, specific inhibitor, aptamer-guided therapy or other methods may enhance the chemosensitivity of OC. The recent advances in EMT and CSCs have unlocked a new avenue for chemosensitivity research. Elucidating the role of EMT and CSCs in the cancer cells' response to chemotherapy will enhance our understanding of OC recurrence after chemodrug treatment, and may direct research towards novel and specific chemosensitizers that target EMT or CSCs. We expect that there will be increased understanding of the intrinsic and extrinsic factors that control the plasticity and maintenance of the EMT/CSC state (e.g. expression factors, miRNA expression, PTMs of molecules that control stem cell fate and niche factors that control stem cell renewal) in OC research.

Since EMT and CSCs have both been implicated in tumourigenesis and chemoresistance, it is critical to examine both populations and determine their phenotypic expression in human OC recurrent tissues in order to develop strategies to target these populations using targeting therapy. Therefore, further evaluation of EMT/CSC markers in OC tissues and investigation on how these cells can be targeted by novel treatment modalities might lead to more effective eradication of OC relapse.

EMT and CSC markers could be also used as important biomarkers for chemotherapy response and prognosis. If these biomarkers for predicting the treatment response of individual OC patient and potential targets for chemosensitization are further validated, it will achieve a more favorable therapeutic ratio in clinics. In addition, future personalized medicine for OC therapy will be developed from EMT and CSC research.

Studying the mechanisms of OC chemoresistance and metastasis is important for novel therapies. Chemoresistant mouse models or other contemporary models such as patients-derived xenografts (PDXs) or explanted human tissues from chemoresistant OC patients should be considered for new drug screening and testing. Combination of chemotherapy with novel agents targeting EMT/CSCs for improvement of chemosensitivity has demonstrated promise in preclinical studies on OC cell lines and animal models. However, only very limited data could be found in Phase I trials. These combination approaches should be further explored in clinical trials to validate their clinical effects in OC therapy.
